# Immigration, political trust, and Brexit – Testing an aversion amplification hypothesis

**DOI:** 10.1111/bjso.12233

**Published:** 2018-01-10

**Authors:** Dominic Abrams, Giovanni A. Travaglino

**Affiliations:** ^1^ Centre for the Study of Group Processes School of Psychology University of Kent Canterbury UK

**Keywords:** voting behaviour, immigration, political trust, threat, European identity

## Abstract

A few weeks prior to the EU referendum (23rd June 2016) two broadly representative samples of the electorate were drawn in Kent (the south‐east of England, *N *=* *1,001) and Scotland (*N *=* *1,088) for online surveys that measured their trust in politicians, concerns about acceptable levels of immigration, threat from immigration, European identification, and voting intention. We tested an aversion amplification hypothesis that the impact of immigration concerns on threat and identification would be amplified when political trust was low. We hypothesized that the effect of aversion amplification on voting intentions would be mediated first by perceived threat from immigration, and then by (dis) identification with Europe. Results in both samples were consistent with this hypothesis and suggest that voters were most likely to reject the political status quo (choose Brexit) when concerns that immigration levels were too high were combined with a low level of trust in politicians.

## Background

On 23rd June 2016, a referendum was held in the United Kingdom in which 52% voted to leave the European Union and 48% voted to remain. The immediate consequence was a precipitous drop in the value of the pound, followed by a great deal of confusion and uncertainty as the ruling Conservative Party's leader David Cameron resigned, and a search for a new leader commenced. This historic vote has raised a large number of questions about the state of the United Kingdom, the rise of populism, and the idea of nations and nationhood. It also prompted a number of speculations on what motivated people to vote to leave the EU.

People's voting choices do not necessarily reflect rational decision‐making (Sears, Lau, Tyler, & Allen, 1980). Although issues such as immigration may be influential (Mughan & Paxton, 2006), the extent of their influence varies because perceptions of threat and attitudes to minority groups are partially shaped by other contextual factors or contemporary events (Abrams, Houston, Van de Vyver, & Vasiljevic, 2015). Political commentators and media speculators have proposed at least two reasons for the support attracted by the Leave Campaign. One of these is that many sections of the electorate were primarily concerned over apparently unprecedently high uncontrolled levels of immigration (see Ipsos MORI, 2016). This is the ‘immigration’ explanation. The other is a failure of trust in politics. Politicians, almost by default, are vulnerable to being mistrusted because their power position means they quite often fail in terms of their ability to reassure the electorate they will serve the common good rather than personal interests (see Fiske & Durante, 2014). This research tests these two explanations using two large samples from Kent and Scotland.

### The context: Key issues in the UK referendum campaigns

#### Immigration

In the EU referendum, an important focus of the campaigns was how to deal with an apparently too high level of immigration. In 2010, the then Home Secretary, Theresa May, committed to reduce net immigration to ‘tens of thousands’ per year. By 2015, the numbers had peaked at 336,000, and by the time of the EU referendum remained at over 320,000, despite repeated government pledges to stick to the ‘tens of thousands’ target (Eaton, 2016). However, there was considerable debate about the meaning of these figures because the majority of immigrants were from outside the EU, and large numbers were in the United Kingdom as students or seasonal workers. While immigration levels created pressures on housing and services, it also contributed substantially to the UK economy. Therefore, a key question for political commentators was how people's concerns about immigration would be connected to their intention to vote to leave or remain in the EU.

#### Trust

A second major theme referred to a breakdown of trust in politics, leading to a revolt against the established political elites (e.g., Mason, 2016; Stanley, 2016). Political trust is a crucial aspect of the relationship between citizens and institutions (Hosking, 2014; Levi & Stoker, 2000; Mishler & Rose, 2001). In general terms, political trust refers to the faith people have in their government (Citrin & Muste, 1999). Low levels of trust imply that individuals should devise ways to protect their own interests autonomously.

Across the United Kingdom as a whole, since 2010 there had already been evidence of a haemorrhaging of electoral support both from traditional Conservative (right wing) and Labour (left wing) voters towards the overtly anti‐establishment UK Independence Party (UKIP). Although Conservative supporters were more likely to vote Leave, a substantial proportion of Labour supporters did too.

Despite the fact that the ‘first‐pass‐the‐post’ electoral system substantially limited UKIP's chances of gaining seats in parliament, UKIP played a prominent role in the Leave campaign, both by fostering mistrust against the British political establishment and by attacking the European Union. Specifically, the Leave campaign repeatedly highlighted that ‘unelected bureaucrats in Brussels’ were controlling UK laws and policies and that the UK's elected politicians were therefore insufficiently accountable over immigration policy.

In the context of the EU referendum, distrust against the political establishment may have been further increased by the apparent ambivalence towards Europe expressed by Remain campaigners. This might have undermined confidence that the levels of immigration would be addressed by the existing political structure.

#### Regional context

In the present research, we examine unique evidence from two separate population samples of 1,000 eligible voters each from Kent and Scotland, to test the *immigration* and *political trust* explanations about why people voted to leave. UKIP and the Leave campaign gained particularly strong support in some of the counties with ports facing Europe in the south of England, especially the county of Kent. The ongoing pressure of the ‘Jungle’ (a refugee and asylum seeker camp at Calais), frequent stories that immigrants were being illegally transported in container trucks and discovered at Folkestone and Dover and on the arterial transport routes through Kent towards London, made the immigration issue particularly salient (Ibrahim, 2011). In contrast, the political climate was different in Scotland, where the Scottish National Party held all but a handful of seats in the Scottish Parliament, and where a majority of voters favoured remaining in the EU.

Psychological research has yet to provide an empirical test of the relative support for the immigration and trust explanations, or indeed how they combine. Political scientists have argued strongly that it is crucial to examine the role of trust using comparative analyses, ideally with the same methodology and measurement but from different regional contexts (Eder, Mochmann, & Quandt, 2015). This is precisely the approach taken in the present paper. Although we expect mean level differences on the measures between the two samples, we regard them as conceptual replications because the psychological processes leading to voting intentions should be consistent despite these different contexts. Thus, to the extent that the contexts differ but the same underlying processes are at work we should find differences in means but replication of the relationships among variables in the two contexts.

### The aversion amplification hypothesis

Political science has tended to treat trust primarily as a contributing independent or mediating variable, but there is no unifying theory about its role in the political process (see Eder *et al*., 2015). However, Eder *et al*. argue, based on Hirschman's (1970) work on exit, voice and loyalty, that trust should be considered as a ‘moderator variable in the relationship between dissatisfaction and participation choices’ (p. 8) when there are particular political issues over which uncertainty exists. Given the dearth of formal hypotheses about such moderation, the present work drew on social psychological perspectives to predict the likely moderating function of trust.

We contend that although the trust and concerns about immigration could each contribute to voting intentions, they were likely to produce a catalytic effect together. First, previous research has shown that trust may have a strong impact on the way individuals appraise their social context. For example, research on risk management indicates that individuals who perceive lower trust in institutions or their government also report a more amplified perception of risk, and are less likely to follow advice to mitigate it (Herreros & Criado, 2009). Moreover, Arndt (2015) argues that proportional voting opportunities (such as a referendum) in a usually majoritarian system (as in the United Kingdom) lead to higher participation among those who have little trust or attachment to political parties. This is because the voting system offers a route to ‘exit’ from prior political choices without having to exit from the political process as a whole, thus offering ‘voice’ to the overall society. This account may to capture the motivation of some Leave voters in the EU referendum and underlines the important role of trust in driving people's voting choices.

We propose a process of *aversion amplification,* whereby the combination of concerns that levels of immigration are too high and low trust in politicians may have propelled some people to vote to leave the EU. We also expect that the psychological vehicles for this effect are stronger perceived threat from immigrants, and identification. Specifically, the combination of higher concerns and lower trust seemed likely to promote heightened perceptions of threat from immigration and thence to undermine identification with the superordinate category, as European.

#### Threat from immigration

A good deal of research has examined psychological threat from immigration. According to integrated threat theory (Stephan & Stephan, 2000), people feel threatened by outgroups when they believe that these groups impinge negatively on aspects of life such as employment prospects (economic), their health and opportunities (material), and their culture and way of life (symbolic threat). Concerns about immigration do not automatically translate into the experience of threat. Previous research has shown that levels of perceived threat may be activated more strongly among some individuals than others, depending on contextual factors (Abrams & Eller, 2017; Van de Vyver, Houston, Abrams, & Vasiljevic, 2016). In this research, we test the idea that concerns about immigration activate threat more strongly when trust in politicians is low (i.e., aversion amplification). We also hypothesize that higher threat will motivate people to form stronger intentions to avoid the source of the threat. In the referendum, this would mean leaving the EU as a means of controlling immigration.

#### European identity

Previous research has shown that social identity can be a proximal mediator between perceptions of social reality (such as deprivation or injustice) and political separatism (Grant, Bennett, & Abrams, 2017). Previous research also shows that group identification creates a psychological anchorage that inhibits turnover among group members (Ng, 2015; Randsley de Moura, Abrams, Retter, Gunnarsdottir, & Ando, 2009). Conversely, weaker identification with a group may release such constraints because the self‐concept is no longer implicated in the group membership. Therefore, we measured people's identification as European and predict that the less strongly people identify with the EU the more likely they should be to vote to leave it.

Just as there may be a range of influences on threat, a number of factors might influence identification. However, we expect that identification will mediate the impact of trust, perceptions of immigration levels, and threat on intentions to vote leave. The EU was depicted throughout the referendum campaign as a superordinate body imposing norms on the national subgroup, resulting in high levels of uncontrolled immigration in the United Kingdom. It would therefore make sense that aversion amplification should stimulate disidentification with Europe and that identification should be the most proximal predictor of people's intentions to vote to remain or leave.

## Method

Parallel surveys were conducted in Kent and in Scotland through Qualtrics Panels. The panels were designed to reflect a representative section of the voting age population across areas of residence and to match the standard of 95% confidence with an error rate of <5% that is conventional for population surveys (power = .99 to detect a small effect with alpha = .001). The surveys were completed online and participants received a small fee (£5). The authors had no role in data collection. The surveys commenced 3 weeks prior to the EU referendum and closed before the referendum date. Some sections of the surveys concerned other issues unique to the particular region. In the present analyses, we also control for a number of potentially relevant variables including political conservatism, age, and gender. We did not measure socio‐economic status directly but had two indicators in the form of measures of educational level and home ownership. These measures were included to ensure that the relationships among the focal variables cannot be explained as spurious effects due to the associations with demographic variables. Results from the focal variables are not altered when covariates are omitted.

### Procedure and measures

The surveys underwent a soft launch to check for issues such as timing and interpretability, although none arose. In Kent (or Scotland), the research was introduced as ‘a survey of people living in Kent (Scotland)’ focusing on views about the EU referendum. Participants read a consent form informing them that all data would remain anonymous and that no one other than the research team would have access to the responses. They were asked to create a unique personal code and were informed they could use it to withdraw their data. To be eligible for participation, respondents first had to confirm that they lived in Kent (Scotland), and to identify their city, town, or village. Participants were also asked to report their age and gender. At the end of the survey, they were asked to indicate their highest educational qualification, and whether they (in Kent) or their family owned/were buying rather than renting their home.

#### Concerns about immigration

This was measured using a standard item from previous UK surveys (Bynner *et al*., 1993; cf. Card, Dustmann, & Preston, 2005). Participants were asked to what extent they strongly disagreed (1) or strongly agreed (5) that ‘No more foreigners wanting to live in this country should be allowed in’. This item was intended to tap individuals’ concerns that immigration levels in the country were too high.

#### Political trust

This was measured using two items based on more extended measures of political trust (e.g., Boukes & Boomgaarden, 2016): ‘Most members of UK parliament are honest’; ‘Most members of the UK parliament can be trusted to defend the interests of the area that elected them (their constituency) above all else’. Responses were on a scale from Strongly Disagree (1) to Strongly Agree (5). Within both regions, the two trust in politicians items were highly correlated, *r*_Kent_(814) = .58, *p *<* *.001; *r*_Scotland_(899) = .66, *p *<* *.001, and were therefore averaged to form a composite score.

#### Threat

Three items measured participants’ sense of threat. These items were drawn from comparable items in prior representative surveys in the United Kingdom and EU (e.g., Abrams & Houston, 2006; Card *et al*., 2005). Symbolic threat was measured with the item ‘Immigrants are people who come to settle in Britain. How do you think immigrants affect the customs, traditions or general way of life of other people in Britain? Do you think they make things worse or better?’ Realistic threat was measured with the item: ‘How do you think immigrants affect things like the safety, security, or health of other people in Britain? Do you think they make things worse or better?’ Both items responded to using a scale from much worse (1) to much better (5). Economic threat was measured using the item: ‘People who come to live in this country generally work and pay taxes at some points in their lives. They also use health and welfare services. On balance, do you think that immigrants in Britain take out more from the economy than they put in, or not?’ This item was responded to on a scale from ‘take out a lot more than they put in’ (1), to ‘put in a lot more than they take out’ (5). Scores on these items were reverse scored so that a higher score represented greater threat. As the three threat items formed a reliable scale within both regions (Cronbach's Alpha = .91 in Kent and .88 in Scotland), the items were averaged to provide a composite score.

#### European identification

Participants were asked to what extent (1 = *not at all*, 7 = *extremely*) they endorsed the statements: ‘Being European is important to me’; ‘I feel European’. The two items were highly correlated within both regions, *r*_Kent_(814) = .87, *p *<* *.001; *r*_Scotland_(899) = .82, *p *<* *.001, and were averaged to form a composite score.

#### Voting intention

Participants were informed: The 23rd June 2016 referendum on Europe will ask: ‘Should the United Kingdom remain a member of the European Union or leave the European Union?’ How do you intend to vote? Response options were *Remain a member of the European Union* (1); *Leave the European Union* (2); *Unsure/undecided* (18); *Would not vote* (19). Because this research focuses on comparing those who would vote remain versus leave, for the purposes of most analyses the variable was recoded as 0 (remain) and 1 (leave). Participants who indicated they were unsure or would not vote responses were excluded from the analyses.

#### Political orientation

To measure people's position on a liberal‐conservative continuum, given that ‘left’ corresponds to politically liberal attitudes in most western countries, participants were instructed: Many people think of political attitudes being on the ‘left’ or ‘right’. This is a scale stretching from the Left to the Right. When you think of your own political attitudes, where would you place yourself? Responses were on a scale from Left (1) to Right (7).

## Results

### Data analytic strategy

We first describe the basic demographics and voting intentions of the two samples, then conduct parallel analyses using the two different samples, examining all those who expressed an intention to vote leave or remain (81.4% in Kent, 82.8% in Scotland). Aside from the regional and demographic differences, we regard one sample as a conceptual replication of the other for the purposes of testing the psychological processes.

### Sample characteristics

#### Kent

In the Kent sample (*N *=* *1,001), 50% were female. The mean age was 47.2 years (*SD* = 14.89). Thirty eight point one per cent indicated they would vote remain, 43.3% would vote leave, 15.6% were unsure, and 3% said they would not vote. Of those who said they would vote, 46.8% preferred Remain and 53.2% preferred Leave (the final referendum results were 41% Remain and 59% Leave, reflecting the acceleration of support for Leave immediately prior to the vote). In terms of political orientation, 23.5% of the participants described themselves towards the politically liberal (‘left’) end of the scale, 42% as neutral, and 34.5% towards the politically conservative (‘right’) end of the scale.

Fifty‐nine point one per cent had a higher education qualification, 21.9% had post 16 qualifications, and 25.3% had only basic school qualifications (up to GCSE/16‐year‐old levels). 68.8% owned or were buying their home, and 27.3% were in rental accommodation. Ninety‐one point eight per cent of the sample described themselves as White/White British, and the remaining participants described themselves predominantly as mixed heritage (2.6%), Asian/Asian British (3.6%), or Black/African/Caribbean/Black British (1.3%).

#### Scotland

In the Scottish sample (*N *=* *1,088), 50.2% were female. The mean age was 48.95 years (*SD* = 15.56). Fifty point seven per cent indicated they would vote remain, 32.2% would vote leave, 15.7% were unsure, and 1.5% said they would not vote. Of those who said they would vote, 61.2% preferred Remain and 38.8% preferred Leave (the referendum vote was 62% remain and 38% leave). Politically, 30.5% described themselves as liberal, 42.9% as neutral, and 24.7% described themselves as conservative. Fifty‐five point nine per cent had a higher education qualification, 21.8% had post 16 qualifications, and 20% had basic school qualifications (up to GCSE/16‐year‐old levels). Sixty six point one per cent owned or were buying their home, and 18.2% were in rental accommodation. Sample selection required that all participants described themselves Scottish. Owing to an oversight ethnicity was not measured, but demographically Scottish people in Scotland are predominantly defined as White British (Scottish Census).

As shown in Table 1, in Kent and in Scotland, Remain voters were significantly more likely to have a higher degree, to be younger and tended to own their home, but did not differ in terms of gender or ethnicity (measured in Kent).

**Table 1:** Means and standard deviations for variables among respondents intending to vote Remain and Leave in the UK EU referendum, 23rd June 2016

Sample	Variable	Remain Mean (*SD*)	Leave Mean (*SD*)	*t* or χ^2^ (*df*)	95% CI
Kent	Immigration concerns	2.36 (1.13)	3.49 (1.25)	13.44 (811)***	1.29, 0.96
	Trust in politicians	3.02 (0.95)	2.64 (0.94)	5.71 (812)***	0.25, 0.51
	Threat	2.87 (0.79)	4.00 (0.83)	19.71 (811)***	1.02, 1.24
	Euro‐identification	4.43 (1.61)	2.16 (1.56)	20.38 (812)***	2.05, 2.49
	Political orientation	3.87 (1.44)	4.47 (1.23)	6.48 (812) ***	0.79, 0.42
	Age	43.88 (15.14)	50.34 (14.00)	9.27 (4)	8.47, 4.45
	% Female	47.2	46.9	0.01 (1)	
	% higher degree	57.2	43.9	14.42 (1)***	
	% home ownership	76.3	70.2	3.73 (1)	
	% White/White British	89.3	93.1	3.56 (1)	
Scotland	Immigration concerns	2.41 (1.13)	3.48 (1.25)	13.25 (897) ***	1.23, 0.91
	Trust in politicians	3.07 (0.91)	2.72 (0.93)	5.68 (897)***	0.24, 0.48
	Threat	2.95 (.83)	3.84 (.87)	15.36 (897)***	78, 1.01
	Euro‐identification	4.33 (1.62)	2.18 (1.57)	19.71 (897) ***	1.94, 2.37
	Political orientation	3.72 (1.38)	4.19 (1.26)	5.10 (897)***	0.65, 0.29
	Age	47.94 (15.90)	50.58 (14.89)	2.43 (863)*	4.77, 0.51
	% Female	47.1	46.1	0.79 (1)	
	% higher degree	65.5	45.0	36.61 (1) ***	
	% family home ownership	82.5	79.5	6.58 (1)*	

Notes

More positive values for each variable have the following meaning: Vote (leave); Immigration concerns (higher); Trust (higher); Threat (higher); Identity (higher); Left–Right political orientation (right); Gender (female); Age (older); Education (higher); Property (greater ownership). In Kent, home ownership was own. In Scotland, home ownership was family (self or parent). *T*‐tests are used to test for differences for interval variables, chi‐square tests for categorical variables.

***p <* *.001; *p <* *.05.

### Aversion amplification hypothesis

Table 2 shows Pearson product‐moment correlations among variables (point biserial correlations in the case of binary variables). Voting intention was significantly related to all measures in the expected directions consistent with the mean differences observed in Table 1. Importantly from the point of view of the aversion amplification hypothesis, trust and concerns about immigration levels were not significantly related to one another in either Kent or Scotland. Moreover, as expected, the two variables that were most highly related to voting intention in both countries were European identification and threat.

**Table 2:** Correlations among variables in the Kent sample (above diagonal) and Scotland sample (below diagonal)

	1 Vote	2 Immigration concerns	3 Trust	4 Threat	5 Identity	6 Left–Right	7 Gender	8 Age	9 Education	10 Property
1. Vote		.43***	−.20***	.57***	−.58***	.22***	−.01	.22***	−.13***	−.07
2. Immigration Concern	.40***		−.02	.49***	−.27***	.31***	.04	.11**	−.12***	−.10**
3. Trust in politicians	−.19***	−.04		−.31***	.24***	.19***	−.13***	.07	.12***	.10**
4. Threat	.46***	.50***	−.33***		−.60***	.19***	.07	.26***	−.24***	−.04
5. European Identity	−.55***	−.28***	.31***	−.50***		−.14***	−.01	−.23***	.21***	.07*
6. Left–Right	.17***	.31***	.16***	.17***	−.06		−.10**	.06	.01	.13***
7. Gender	−.01	.02	−.07*	.05	−.01	.02		−.14***	−.01	−.02
8. Age	.08*	−.02	.07*	.11***	−.11***	−.01	−.22***		−.10**	.18***
9. Education	−.20***	−.25***	.12***	−.28***	.25***	−.01	.03	−.15***		.19***
10. Property	−.09**	−.08*	.08*	−.05	.05	.05	−.01	.07	.12***	

More positive values for each variable have the following meaning: Vote (leave); Immigration concerns (too high); Trust (higher); Threat (higher); Identity (higher); Left–Right political orientation (right); Gender (female); Age (older); Education (higher); Property (greater ownership).

***p <* *.001; **p <* *.01; *p <* *.05.

To establish the viability of the aversion amplification hypothesis, we first conducted simple moderation tests using SPSS PROCESS Model 1 (Hayes, 2012) with 5,000 bootstraps. Immigration level and political trust were the independent variables.

In Kent (*N *=* *813), the immigration concerns × political trust interaction term was significant for threat (*b *=* *0.12; *SE* = .02, *t *=* *5.86, *p *<* *.001, 95% CI [0.08, 0.16]); identification (*b *=* *0.10; *SE* = .05, *t *=* *2.13, *p *=* *.034, 95% CI [0.01, 0.19]); and vote (*b *=* *−0.14; *SE* = .07, *Z *=* *−2.14, *p *=* *.032, 95% CI [−0.27, −0.01]).

In Scotland (*N *=* *899), the interaction term was significant for threat (*b *=* *0.14; *SE* = .02, *t *=* *7.52, *p *<* *.001, 95% CI [0.10, 0.17]); identification (*b *=* *0.20; *SE* = .04, *t *=* *4.84, *p *<* *.001, 95% CI [0.12, 0.28]) but not vote (*b *=* *−0.08; *SE* = .06, *Z *=* *−1.27, *p *=* *.20, 95% CI [−0.20, 0.04]).

**Figure 1:**
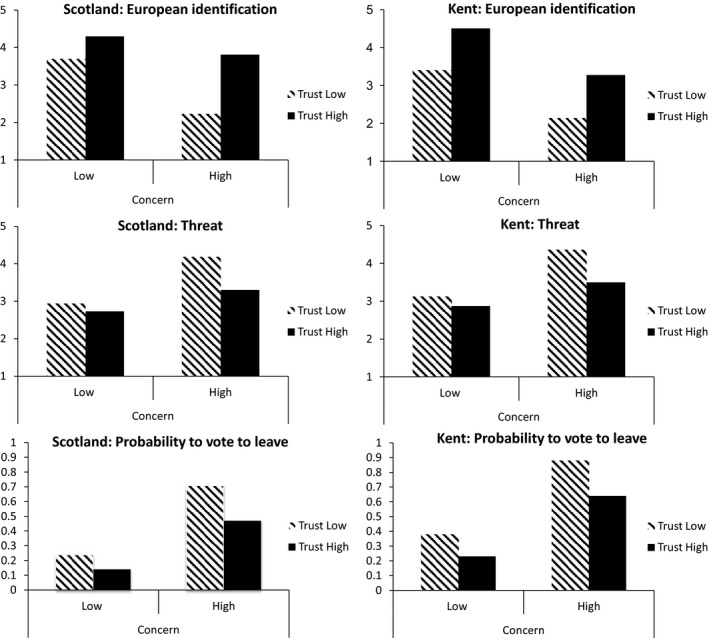
Interactive effect of political trust and immigration concerns on European identification, perceived threats, and intention to vote to leave the European union.

Figure 1 shows a consistent pattern across Kent and Scotland. Threat was highest, European identification was lowest, and intention to vote leave was strongest when people believed immigration level was too high and their political trust was low.

### Indirect effects of aversion amplification

**Figure 2:**
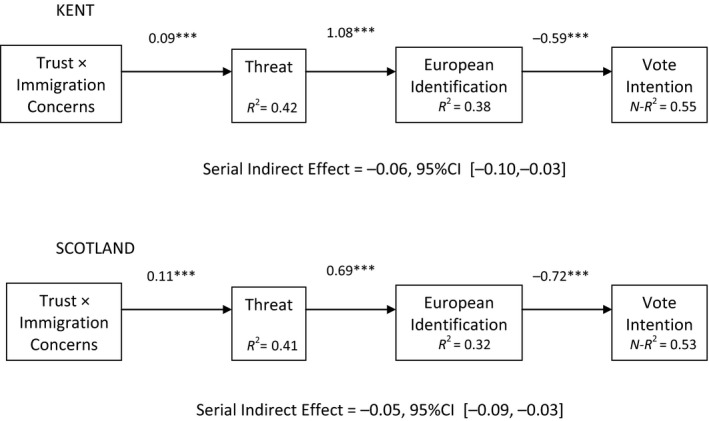
Serial indirect effect of aversion amplification on voting intention in Kent and in Scotland (***p <* *.001).

We proceeded to test the hypothesis that threat and European identification potentially mediate the effects of aversion amplification on voting preference, using logistic binary regression and PROCESS Model 6 (moderated serial mediation) with 10,000 Bootstraps (see Hayes, 2015; p. 12). This enables a formal test of the mediation of the interaction term through the first mediator, the second mediator, and serially. In practical steps, the first main effect is specified as the independent variable and the second main effect and interaction term are entered as covariates along with the five control covariates. The control covariates – political orientation, gender, age, education level, and home ownership – were included on the basis of their correlations with threat and identification. Our model specified that the interactive effect of immigration concerns and political trust should operate indirectly first via threat and then via European identification, to predict voting intention (see Figure 2). The syntax described in Hayes (2015) generates a further data matrix from which the confidence intervals for the moderated mediation are derived.

#### Kent

The overall model significantly predicted voting intention, *N *=* *782, Nagelkerke *R* ^2^ = .585, −2LL = 629.91, Model LL = 450.97, *p *<* *.0001. Three of the covariates also had significant effects. These were political orientation (*b *=* *0.33, *SE* = .07, *Z *=* *4.69, *p *<* *.001, 95% CI [0.19, 0.47]), age (*b *=* *0.04, *SE* = .01, *Z *=* *5.58, *p *<* *.001, 95% CI [0.02, 0.05]), and home ownership (*b *=* *−0.47, *SE* = .21, *Z *=* *2.29, *p *=* *.022, 95% CI [−0.88, −0.07]). Respondents who were more right wing, older and less likely to own their own home were more likely to vote to leave.

An inspection of the model revealed significant total indirect effects of the interaction term (*b *=* *−0.09, *SE* = .047, 95% CI [−0.19, −0.01]), and a non‐significant direct effect (*b *=* *0.04, *SE *= .09, *Z *=* *0.48, *p *=* *.63, 95% CI [−0.14, 0.23]). There was a significant indirect effect of the interaction via threat (*b *=* *0.07, *SE* = .03, 95% CI [−0.13, −0.03]) and a significant sequential mediation through threat and then European identification (*b *=* *−0.06, S*E *= .02, 95% CI [−0.10, −0.03]). The mediation solely through the proximal mediator (identification) was not significant (*b *=* *0.04, *SE *= .02, 95% CI [−0.01, 0.09]). Contrasts also showed that the latter indirect effect was smaller than the indirect path solely through threat (contrast coefficient = −.11, *SE* = .03, 95% CI [−0.18, −0.05]) or sequentially through threat and then identification (contrast coefficient = −.10, *SE* = .03, 95% CI [−0.16, −0.05]).

#### Scotland

The overall model significantly predicted voting intention, *N *=* *731, Nagelkerke *R* ^2^ = .52, −2LL = 618.47, Model LL = 357.34, *p *<* *.0001. Reduction in *N* was owing to missing data on home ownership. As this had been a significant covariate in the Kent sample, it was decided to retain this covariate in the Scotland sample, although in fact none of the covariates had significant effects, and results were the same when this covariate was excluded. An inspection of the model revealed significant total indirect effects (*b *=* *−0.17, *SE* = .04, 95% CI [−0.25, −0.08]) and a significant direct effect (*b *=* *0.28, *SE* = .09, *Z *=* *3.26, *p *=* *.001, 95% CI [0.11, 0.45]). There was a significant indirect effect of the interaction via threat (*b *=* *−0.04, *SE* = .02, 95% CI [−0.09, −0.01]) and a significant sequential mediation through threat and then European identification (*b *=* *−0.05, *SE* = .02, 95% CI [−0.09, −0.03]). The mediation solely through the proximal mediator (identification) was also significant (*b *=* *−0.07, *SE* = .03, 95% CI [−0.13, −0.01]). The size of these three indirect paths did not differ significantly.

#### Reverse mediation tests

Across samples, we checked the plausibility of the reverse mediation sequence, namely that aversion amplification first affected European identification and then threat (thus treating threat as the proximal predictor of voting intention). In Kent, this reverse sequential mediation was non‐significant (*b *=* *−0.01, *SE* = .01, 95% CI [−0.02, 0.01]). In Scotland, the reverse sequential mediation was significant (*b *=* *−0.01, *SE* = .005, 95% CI [−0.02, −0.002]). However, the coefficient for the indirect effect via only European identification (−0.12) was significantly larger than that via both identification then threat (−0.01) (contrast coefficient = −0.11, *SE* = .03, 95% CI [−0.18, −0.05]) and via only threat (−0.03) (contrast coefficient = −.09, *SE* = .04, 95% CI [−0.17, −0.02]). Therefore, the evidence is more consistent with the idea that European identification is likely to be a most proximal mediator.

## Discussion

The evidence for this study provides a unique insight into the social psychology of Brexit in two key regions of the United Kingdom, Kent and Scotland. The two surveys were conducted shortly prior to the actual referendum, so we are reassured that they were adequately representative of opinion. Indeed, the patterns of voting intentions were quite consistent with the actual referendum results. Given that the ‘Leave’ vote won by only 4%, even quite small influences on voting were extremely important. We used the data to test a conceptual model explaining individuals’ propensity to vote to leave. Results supported the model in these two distinct parts of the United Kingdom. The two data sets therefore provide conceptual replications, which highlight the robustness of these findings. At the same time, they offer interesting empirical comparisons.

We proposed that concerns about whether immigration levels are too high may be more likely to generate symbolic, economic, and realistic forms of immigration threat and disidentification with Europe under certain conditions. This argument is based on the idea that prejudice and acts of exclusion can have a variety of roots, ranging from personal discontent and mistrust (malign antipathy), to rivalrous forms of cohesion when intergroup divisions are more salient (Abrams & Vasiljevic, 2014; Hosking, 2014). The referendum may have stimulated both states and thereby stimulated rejection of the EU.

Political commentators have focused on two explanations for the success of the Leave campaign. One explanation is about perceptions of immigration being out of control. The second emphasizes the breakdown in political trust and consequent rise in populism. For example, support for Brexit was stronger in those areas of the country which felt left behind by the political establishment, or that historically repudiated the main parties and politicians in Westminster (Clarke, Goodwin, & Whiteley, 2017). Based on recent theorizing (Arndt, 2015; Eder *et al*., 2015), we contended that trust should act as a moderator variable. Our aversion amplification hypothesis contended that when these two factors combined they would catalyse aversion to the EU. Specifically, we proposed that belief that immigration levels were too high in combination with mistrust of UK politicians would amplify perceived threat from immigration and weaken European identification, thereby mobilizing intentions to vote to leave the European Union – a combination of exit from the status quo but expressing political voice to gain control over the change (cf. Arndt, 2015; see also Travaglino, 2017).

Our choice of context for this research was important. Whereas the Leave campaign and its ‘maverick’ political figureheads in such as Nigel Farage (leader of UKIP) and Boris Johnson (recent Mayor of London) gained particularly strong support in Kent, they were much less well regarded in Scotland, a region dominated more strongly by left‐wing politics and with little affinity to London‐based political figures. The Leave campaign as a whole peddled highly questionable speculations about the benefits of leaving the EU and sought to cast doubt on the veracity of contrary evidence provided by ‘experts’ (predominantly academics and experienced economists – see Mance, 2016). The campaign may even have deliberately adopted a strategy of weakening political trust (cf. Levi & Stoker, 2000; Mishler & Rose, 2001), undermining the faith people had in the UK government. Such messages clearly had some appeal in both regions in the present study. In Kent, the trust message chimed well with constant news stories about illegal immigrants and asylum seekers and criticism that the government was not acting sufficiently to deal with these. In Scotland, the trust messages clearly reinforced perceptions that the Westminster (UK) parliament was not really interested in or concerned with the interests of Scotland. Lower political trust in turn implies that individuals should devise ways to protect their own interests autonomously (Citrin & Muste, 1999). Yet we argued, and found, that the aversion amplification process should be observed similarly in both of these contexts, providing important comparative support for the hypothesis (cf. Eder *et al*., 2015).

We proposed that aversion amplification would affect voting intentions indirectly via two mediators, threat and identification. We also expected that social identity would be the more proximal mediator between perceptions of social reality (such as deprivation or injustice) and political separatism (Grant *et al*., 2017; Randsley de Moura *et al*., 2009). Results supported these hypotheses and indicated that threat was greatest and identification lowest among respondents who were concerned that immigration levels were too high and who had low levels of trust in politicians. The data are also consistent with the idea that people's feelings of threat also indirectly affected voting intentions by reducing their identification with Europe.

### Strengths and limitations

Among the limitations of the present research are the inevitable sacrifice of measurement precision that goes with time and cost constraints of conducting large surveys with the general population. Ideally, we would have used more items for each of the constructs measured in this study to provide more reliable measures and parameter estimates. However, the measures were based on prior research and the relationships found were consistent with previous theory. None of the relationships were so high as to suggest problems of multicollinearity. A different limitation is the specific operationalization of constructs. For example, our measure of concern referred to immigration generally rather than immigration specifically from the EU. This level of measurement was chosen because the Leave campaign generally focussed on all immigration levels (such as a poster showing queues of Syrian refugees) and argued that leaving the EU would enable better control by removing all immigrants’ indirect access via EU countries.

Although our measures of immigration threat were based directly on prior social psychological research, there is rather less consensus or consistency in the use of trust measures in political science research (see extensive analysis by Eder *et al*., 2015), and therefore, we must acknowledge that different types and foci of trust will need to be explored in future research. In particular, it may seem paradoxical that those who expressed lower trust in UK politicians were most willing to remove power from EU politicians, which might indirectly give more power to UK politicians. However, we note that voting to Leave would give the voter more direct influence over the proximal representative in two ways. One was that because most members of parliament were in favour of a Remain position, voting Leave would directly challenge their preference. The second was that leaving the EU would remove an external influence or constraint (from the EU) on UK politicians’ actions, making them more directly accountable to UK citizens. Therefore, voting to Leave was perhaps not so inconsistent with mistrust in UK politicians.

The large samples used in this research offered advantages of greater generalizability and external validity. The data therefore enabled us to test theoretically specified relationships between variables to predict highly consequential voting intentions in the British EU referendum. The evidence makes a unique contribution to public and scientific debates by providing an empirical test of two different accounts of individuals’ intentions to vote leave or remain. Future research should extend this work by investigating individuals’ voting behaviour using other methodologies and approaches, such as experiments or longitudinal methods. We note, however, that while experiments can test the plausibility of causal relationships among variables, they are not a viable method in real elections because it is neither ethical nor legal for scientists to experimentally manipulate people's voting intentions in such contexts. Nonetheless, simulation studies may shed further light on potential causal roles of different variables and more work needs to be done using the full range of different methods available.

Importantly, evidence about the relationships between demographic and covariate measures and the focal analysis variables helps to allay concerns that we are observing spurious relationships. For example, in both samples, consistent with contemporaneous opinion poll data (see Clarke *et al*., 2017) and political commentary, older age, lower education level, and political conservatism were significantly correlated with intention to vote to leave. Yet we observed equally strong or stronger relationships between voting intention and political trust, concern with immigration levels, and the theoretically specified mediators of threat and identification.

The moderated serial mediation tests in both regions supported a model in which the interactive effects of political trust and immigration concerns operated indirectly through their effects on threat and identification. However, we could not completely rule out the possibility that these mediators might be independently relevant rather than necessarily following the sequence that we specified. This seems reasonable given that their strength could be affected by other contemporaneous factors (e.g., terrorism or sport competitions). Therefore, we conclude that both threat and identification are likely mediators of the aversion amplification effect, but that they may well be conduits of other influences too. In Scotland, although the data were more consistent with our hypothesized mediation sequence, we could not completely rule out a reversed sequence in which identification precedes threat. However, considering both samples, we believe the proposed sequence is more parsimonious.

### Conclusions

The present research is the first to investigate systematically and empirically the psychological processes arising from two explanations of why people voted to leave the EU in the 2016 UK referendum. The evidence provides initial support for an *aversion amplification hypothesis*. In the present research, this hypothesis was that intentions to vote leave would be fuelled when threat and lowered superordinate group identification were amplified because immigration numbers were seen as too high and political trust was low. Stated in more general terms the aversion amplification hypothesis makes a theoretical contribution by articulating the implications of particular forms of the relationship between threat and trust. It suggests that psychological resistance to outgroup members (in the present case, immigrants) is amplified when they are seen as posing a problem that the system cannot be trusted to resolve. Psychological resistance is then likely to be expressed through a range of behavioural intentions or actions.

The aversion amplification hypothesis can be translated to other settings. Future research can test how well it accounts for support for non‐establishment politicians such as Donald Trump in the recent US Presidential election. It could also help to explain when and why people engage in disruptive political action (Jost *et al*., 2012), or when they adopt a social change ideology (Grant *et al*., 2017). Intriguingly, aversion amplification seems to capture a state of ‘malign antipathy’ (Abrams & Vasiljevic, 2014). Such state might be potentially volatile, finding its voice particularly when an opportunity arises to kick out at the system, but being otherwise relatively dormant and non‐systematic in its impacts. For some people, opportunities such as the UK's European referendum may act as lightning rods for such antipathy.

We hope that the present work will stimulate new research in a variety of different contexts. For example, in principle, the rhetoric of ‘taking back control’ may be one that also describes some non‐political contexts, such as when prisoners collectively riot; or citizens stage sit‐ins. The amplification hypothesis also hints at the effect of threat can be shaped by other contextual factors. Specifically, it could be amplified by the release of social constraints (absence of normative controls) or other factors. But it could be attenuated if there are more or stronger constraints in place. Attenuating factors might include individual differences in people's willingness to hold or express prejudices (Kunstman, Plant, Zielaskowski, & LaCosse, 2013); positive contact with immigrants (Meleady, Seger, & Vermue, 2017), their desire to uphold certain values behaviourally (Abrams *et al*., 2015), or other normative beliefs (Scheidegger & Staerklé, 2011), or the presence of restrictive laws or regimes (e.g., in Egypt, Turkey or the Soviet Union). These are all interesting avenues for future research.
